# Collision with duplex DNA renders *Escherichia coli* DNA polymerase III holoenzyme susceptible to DNA polymerase IV-mediated polymerase switching on the sliding clamp

**DOI:** 10.1038/s41598-017-13080-1

**Published:** 2017-10-16

**Authors:** Thanh Thi Le, Asako Furukohri, Masahiro Tatsumi-Akiyama, Hisaji Maki

**Affiliations:** Division of Systems Biology, Graduate School of Biological Sciences, Nara Institute of Science and Technology, Ikoma, Nara, 630-0192 Japan

## Abstract

Organisms possess multiple DNA polymerases (Pols) and use each for a different purpose. One of the five Pols in *Escherichia coli*, DNA polymerase IV (Pol IV), encoded by the *dinB* gene, is known to participate in lesion bypass at certain DNA adducts. To understand how cells choose Pols when the replication fork encounters an obstacle on template DNA, the process of polymerase exchange from the primary replicative enzyme DNA polymerase III (Pol III) to Pol IV was studied *in vitro*. Replicating Pol III forming a tight holoenzyme (Pol III HE) with the sliding clamp was challenged by Pol IV on a primed ssDNA template carrying a short inverted repeat. A rapid and lesion-independent switch from Pol III to Pol IV occurred when Pol III HE encountered a hairpin stem duplex, implying that the loss of Pol III-ssDNA contact induces switching to Pol IV. Supporting this idea, mutant Pol III with an increased affinity for ssDNA was more resistant to Pol IV than wild-type Pol III was. We observed that an exchange between Pol III and Pol IV also occurred when Pol III HE collided with primer/template duplex. Our data suggest that Pol III-ssDNA interaction may modulate the susceptibility of Pol III HE to Pol IV-mediated polymerase exchange.

## Introduction

In *Escherichia coli* (*E. coli*), genome DNA is mainly replicated by DNA polymerase III holoenzyme (Pol III HE)^1,2^. During DNA replication a large protein complex, called Pol III*, composed of two core polymerases (each with α, ε and θ subunits) and one DnaX clamp loader (with δ-δ′-τ_2_-γ-χ-ψ subunits), loads the β sliding clamp onto primed template DNA and forms a tight holoenzyme, Pol III HE, with the β clamp at a primer/template junction. The 5′-3′ polymerase subunit (α) and 3′-5′ exonuclease subunit (ε) of the core simultaneously bind to the β clamp to synthesize and proofread DNA, enabling highly processive, extremely fast, and accurate genomic DNA synthesis at the replication fork^3,4^. None of the other four *E.coli* DNA polymerases forms a complex with the clamp loader as tightly as the Pol III core does^5^. They are reported to interact transiently with the β clamp loaded by Pol III*, and to participate in short-patch DNA synthesis, such as Okazaki fragment maturation, DNA repair, and translesion synthesis (TLS)^6,7^.

One of them, DNA polymerase IV (Pol IV), encoded by the *dinB* gene, is a distributive, slow, and error-prone TLS polymerase. Pol IV bypasses certain types of alkylation lesions and N^2^-dG lesions, such as those caused by benzo(a)pyrene, 4-NQO, and NFZ^8,9^. In fact, Pol IV-deficient *E.coli* is moderately sensitive to NFZ and MMS^9,10^. Both *in vivo* and *in vitro* data suggest that Pol IV also participates in restarting the replication at double-stranded DNA break sites^11–13^. Recent genetic data clearly demonstrated that the exchange between Pol III and Pol IV on the β clamp (Pol III-Pol IV switching) is important for cell survival after DNA damage^14^.

On the other hand, there is a long-standing argument for its alternative role in undamaged cells because it is more abundant than other polymerases^15^. Previous *in vivo* studies suggest that Pol IV can access the replication fork even if there is no exogenously induced DNA damage. Overexpression of Pol IV causes a modest increment of mutation rate and highly overexpressed Pol IV inhibits DNA replication strikingly^16–18^. The conserved clamp-binding motif (CBM) of Pol IV is important for the replication inhibition, suggesting that Pol III-Pol IV switching impedes the fork progression. DNA combing data showed that Pol IV slows the fork speed under SOS-induced conditions upregulating the expression of Pol IV ~10-fold^19^.

The molecular mechanism of Pol III-Pol IV switching has been studied biochemically in the past decade. It is reported that Pol IV itself stimulates an exchange between Pol III and Pol IV when Pol III is stalled by nucleotide omission *in vitro* ^20,21^. Reconstituted replication forks stalled at adducts were quickly rescued by Pol IV-mediated Pol III-Pol IV switching and TLS at the fork^22^. Structural study suggests that this polymerase exchange process is mediated by Pol IV binding to the rim of the β clamp even when the CBM binding pocket is occupied by the Pol III core^23^.

Despite the accumulating data implying that Pol IV has the ability for the polymerase switch, it is still unclear how Pol III-Pol IV switching takes place when Pol III forms the holoenzyme complex with the clamp tightly (the lifetime of the holoenzyme complex is longer than 5 min). It is also unclear if the mechanism for Pol III-Pol IV switching is only for damage tolerance or has an as yet unknown function at the moving fork. In a previous report, we observed that Pol IV rapidly disrupts the stable interaction between stalled Pol III* and the β clamp and facilitates the dynamic exchange of polymerases^21^. This Pol IV-dependent Pol III-Pol IV switching is observed when Pol III is stalled by nucleotide omission, and Pol IV at moderate concentrations basically does not interfere with elongating Pol III HE. At particular sites on template M13 ssDNA, however, we observed that Pol IV caused strong signals pausing elongation by Pol III HE. This suggests that as yet unidentified factors stimulate Pol III-Pol IV switching at those sites. Uncovering them will help us understand the molecular mechanism of Pol IV-mediated Pol III-Pol IV switching.

In this study, we identified one factor stimulating Pol III-Pol IV switching on a lesion-free template by using an *in vitro* DNA synthesis assay. We found that the presence of a short hairpin structure on template DNA strongly stimulates Pol III-Pol IV switching during Pol III-catalyzed elongation even if Pol III is capable of continuing DNA synthesis across the hairpin by the strand-displacement activity with the aid of single-stranded DNA binding protein (SSB). The switch from Pol III to Pol IV takes place immediately when Pol III encounters a hairpin stem and starts invading double-stranded DNA (dsDNA). Mutant Pol III_*dnaE173*_ HE, which has an amino acid substitution in the α subunit and exhibits an increased affinity to ssDNA^24,25^, confers resistance to the effect of Pol IV at the hairpin. We also observed that a Pol III collision with a primer/template duplex strongly stimulated switching from Pol III to Pol IV. Our data suggest that Pol III-Pol IV switching takes place when the α subunit loses contact with ssDNA by the presence of duplex DNA ahead of the enzyme. We think that the ssDNA binding of the Pol III core may be the primary factor determining the susceptibility of Pol III HE to Pol IV-mediated polymerase switching.

## Results

### Pol IV interrupts the Pol III-catalyzed primer elongation when Pol III HE encounters the hairpin structure

**Figure 1:**
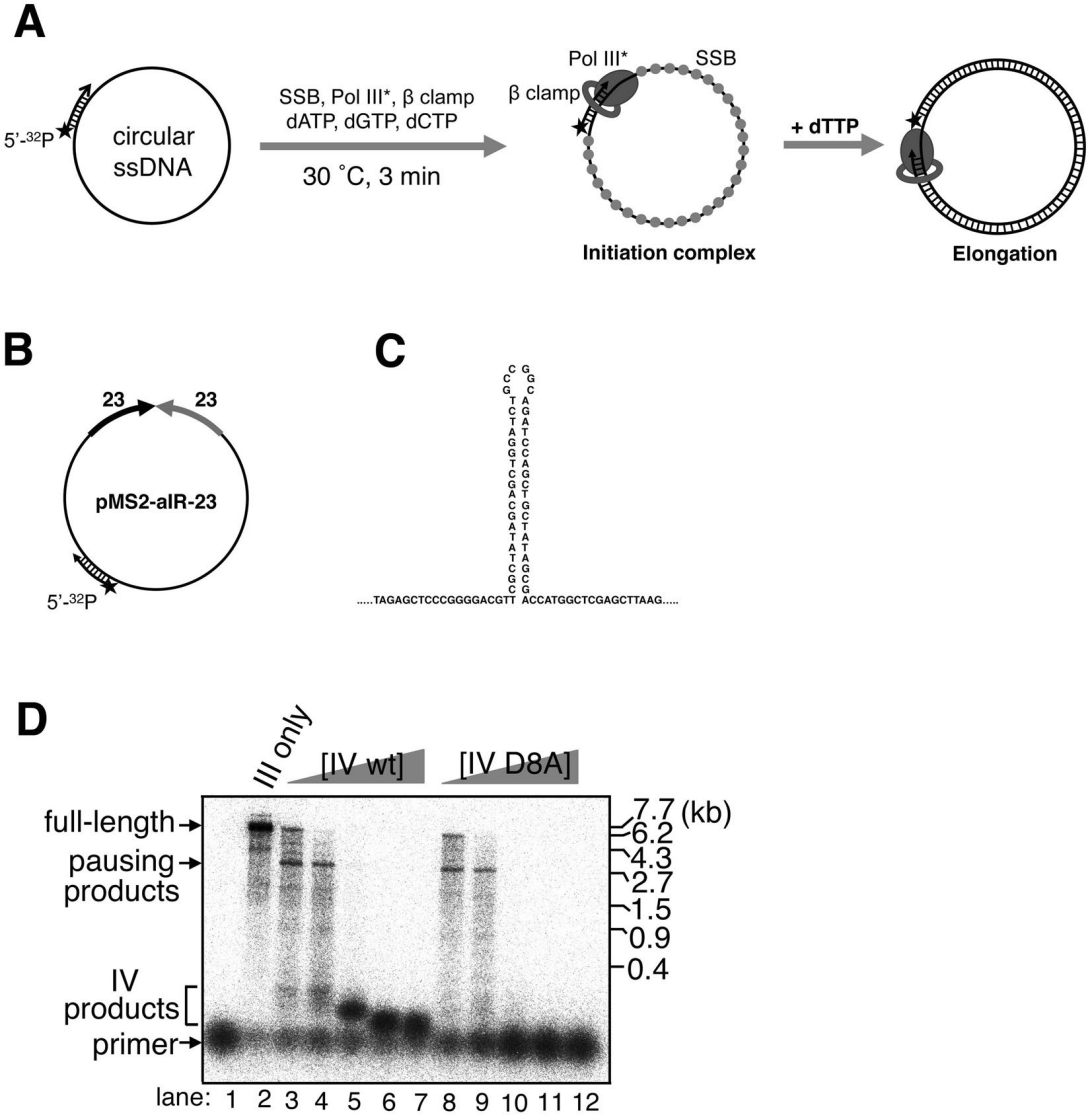
Pol IV interrupts the primer elongation reaction by Pol III on an inverted repeat-containing template DNA. (**A**). Scheme of two-step DNA synthesis assay (also known as burst DNA synthesis assay). (**B**). Template DNA used in the assay. A circular ssDNA carrying a 46-nt inverted repeat was annealed with 5′-^32^P labeled primer locating ~3-kb away from the repeat. A radiolabel is indicated as a black star. (**C**). Predicted hairpin structure formed by 46-nt inverted repeat on the template. (**D**). The effect of Pol IV on the primer elongation reaction by Pol III on primed pMS2-aIR-23 template. The reaction was carried out as in Materials and Methods. Briefly, Pol III HE and template DNA (at the final concentration of 1.2 nM and 1 nM, respectively) were mixed for 3 min. After the formation of the initiation complex, 0, 50, 100, 200, 400, and 800 nM wild-type Pol IV (lanes 2–7, respectively) or Pol IV D8A (lanes 8–12, respectively) was added to the reaction mixture 15 seconds before the start of the chain-elongation process. Replication products after 15-second elongation reaction were analyzed by 0.9% alkaline agarose gel electrophoresis. Template DNA without any treatment was separated as a control (lane 1). Note that quantitative analyses of the results were shown in Supplemental Figure S1 from two independent experiments to test the reproducibility of the result.

A circular 5.2-kb single-stranded DNA (ssDNA) with 46-nt perfect inverted repeat pMS2 (23-nt repeat unit, pMS2^26,27^, renamed pMS2-aIR-23 ssDNA in this work, Fig. 1B and C) was used as a template in the two-step DNA synthesis assay (also called the burst DNA synthesis assay) investigating the effect of Pol IV on Pol III-catalyzed elongation of repetitive sequences. During the initial pre-incubation (3 min), Pol III* loaded the β clamp onto a 5′-^32^P-labeled primer and formed a tight initiation complex at a primer/template junction on a circular ssDNA template. In the presence of three kinds of dNTPs, the resulting Pol III HE (Pol III*-β complex) idled at the primer end. Then a synchronous burst of primer elongation was initiated by adding the fourth dNTP (Fig. 1A). Pol III HE processively elongated the primer without any apparent pausing and produced full-length 5.2-kb products within 15 seconds (Fig. 1D, lane 2). Adding increasing concentrations of Pol IV during the pre-incubation facilitated the polymerase switch between idling Pol III and Pol IV. According to the decrease of 5.2-kb products of Pol III catalysis, an increase of shorter products due to Pol IV catalysis appeared as previously observed (Fig. 1D, lanes 3–7)^21^. Those products were not produced when mutant Pol IV D8A (polymerase-activity deficient mutant^21^) was used, showing that Pol IV rather than Pol III started the burst of primer extension when dTTP was added (Fig. 1D, compare lanes 4–7 and 9–12). Interestingly, Pol IV catalysis products were not clearly observed at the lowest concentration of Pol IV (50 nM, lane 3 in Fig. 1D) but clear pausing products within long products appeared at around the inverted repeat site for both wild-type and mutant Pol IV (~3 kb away from the primer, lanes 3 and 8, Fig. 1D and Supplemental Figure S1), suggesting that Pol IV interrupts Pol III-catalyzed elongation at the inverted repeat.

**Figure 2:**
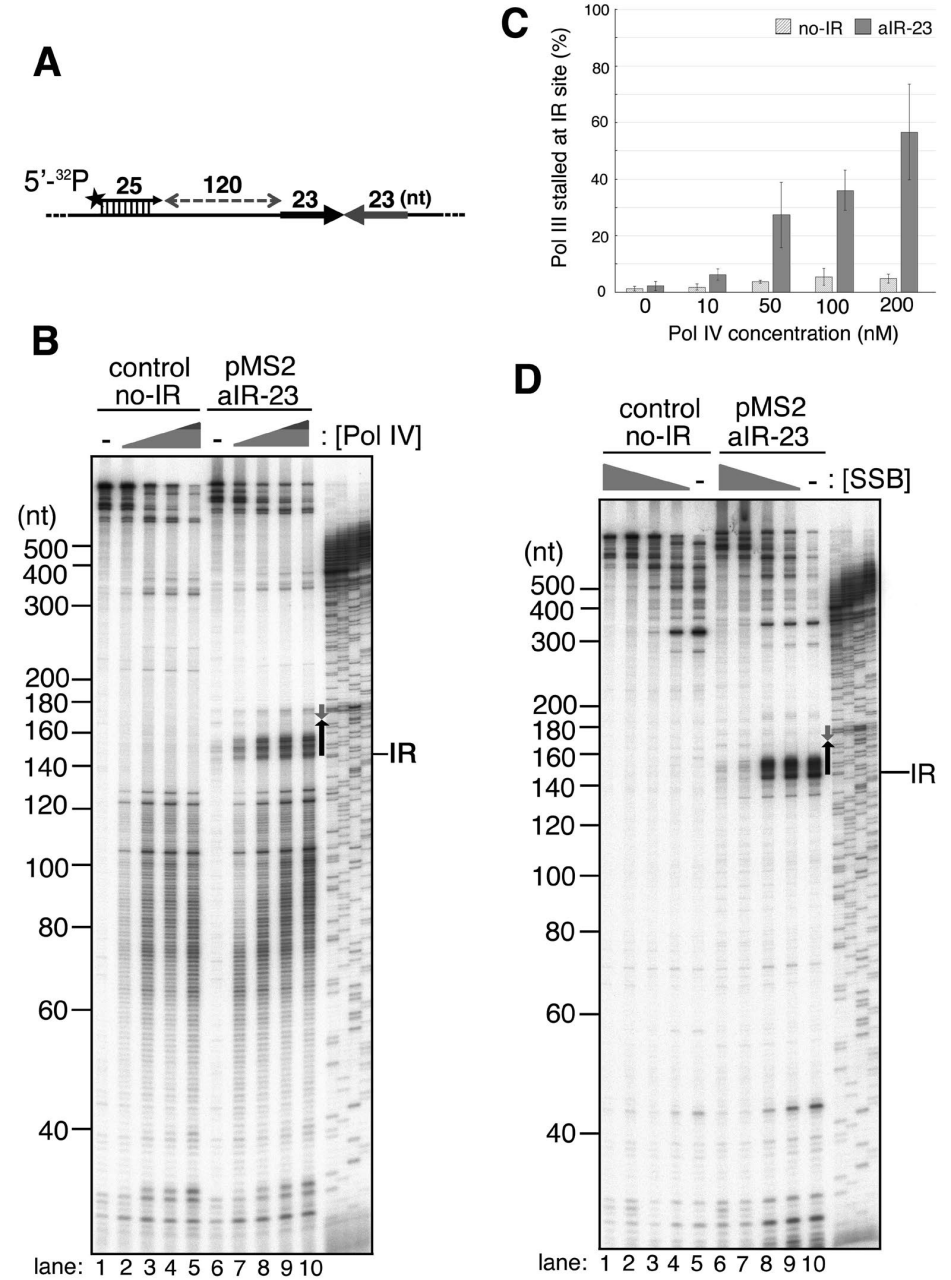
Pol III paused within the inverted repeat sequence in the presence of Pol IV. (**A**). Sketch showing the location of 5′-^32^P labeled primer. Inverted repeat is indicated as black and gray arrows. (**B**). The effect of Pol IV on the primer elongation reaction by Pol III on primed pMS2-aIR-23 template. Wild-type Pol IV (final concentration of 0, 10, 50, 100, and 200 nM) was added together with Pol III HE to the assay using a control, inverted-repeat-free template (pMS2-no-IR, lanes 1–5) or pMS2-aIR-23 template (lanes 6–10). After 3-minute incubation, the elongation reaction was initiated by adding dTTP. Replication products at 10-second incubation were analyzed by 7.5% sequencing gel electrophoresis. Laddering markers representing sequences of pMS2-aIR-23 are shown on the right. Locations of inverted repeats are shown as black and gray arrows in the image. The beginning of inverted repeat in laddering marker is also shown as IR on the right side of the image. (**C**). The effect of Pol IV on ongoing Pol III at the repeat was quantitatively analyzed. The amount of pausing products at the repeat (between ~140 nt and ~191 nt) relative to the amount of products elongated by Pol III (>140 nt) in Fig. 2B were quantified and the percentages were calculated. The average percentages of three independent experiments were shown with standard deviations (SD). Note that short laddering signals (<~140 nt) were omitted as they were Pol IV-catalyzed products. (**D**). DNA synthesis assay in the absence of Pol IV was carried out to analyze the behavior of Pol III HE at the repeat. Replication products at 10-second incubation in the presence of 0.6, 0.3, 0.15, 0.08, and 0 μg of SSB were separated on the sequencing gel. (lanes 1–5: no-IR template; lanes 6–10: aIR-23 template).

To confirm that Pol III pausing occurs at the inverted repeat, we determined the precise pausing site using sequencing gel electrophoresis and another ^32^P-labeled primer located 120-nt away from the repeat (Fig. 2A). As observed in Fig. 1D, along with an increased concentration of Pol IV, short laddering products (<~120 nt) mainly extended by Pol IV were observed for both pMS2-aIR-23 (Fig. 2B, lanes 7–10) and an inverted repeat-free template, pMS2-no-IR (Fig. 2B, lanes 2–5). Within longer products elongated by Pol III HE, strong pausing signals appeared at around the repeat with pMS2-aIR-23 (Fig. 2B, lanes 7–10, the location of the inverted repeat is shown by the gray and black arrows in the image), while they were not observed with the control template (Fig. 2B, lanes 2–5). Even at 10 nM Pol IV (Fig. 2B, lane 7, approximately 10-fold molar excess of Pol IV over Pol III) clear pausing signals appeared at around the repeat and the amount of pausing products increased when Pol IV concentration becomes higher. The analysis of inverted-repeat specific bands indicates that most of the Pol III paused within the upstream half of the repeat (between 145 and 168 nt, shown as a black arrow in Fig. 2B). Although an excess amounts of Pol IV relative to Pol III were added to the reaction (Pol III:Pol IV is 1:8 at 10 nM Pol IV and 1:83 at 100 nM Pol IV), Pol IV is known to be a highly abundant polymerase (330–3,300 nM) and the cellular ratio of these polymerases are similar to that we used in the *in vitro* assay (Pol III:Pol IV is 1:17 in the normal cell and 1:165 in SOS-induced cell^15^). Interestingly, these pausing patterns are similar to those observed when Pol III was hampered by a hairpin structure at the inverted repeat in the absence of SSB (compare the lanes 10 in Fig. 2B and D). It is well-known that SSB helps a polymerase replicate DNA on ssDNA by binding to ssDNA to prevent the formation of secondary structure. At the repeat on pMS2-aIR-23 ssDNA, a short hairpin with a 23-nt dsDNA stem would be formed without SSB (Fig. 1C). In addition, it is reported that the strand-displacement activity of Pol III requires the presence of SSB and interaction with SSB^28^. Thus, decreasing the concentration of SSB results in an increase of Pol III pausing due to the formation of secondary structures on ssDNA (Fig. 2D). In the presence of SSB, most of the Pol III HE can overcome such a short hairpin and naturally formed various structures on ssDNA by its strand-displacement activity (Fig. 2D, lane 6), but in the absence of SSB, Pol III pauses within the hairpin stem (pauses within the upstream half of the repeat, Fig. 2D, lane 10).

**Figure 3:**
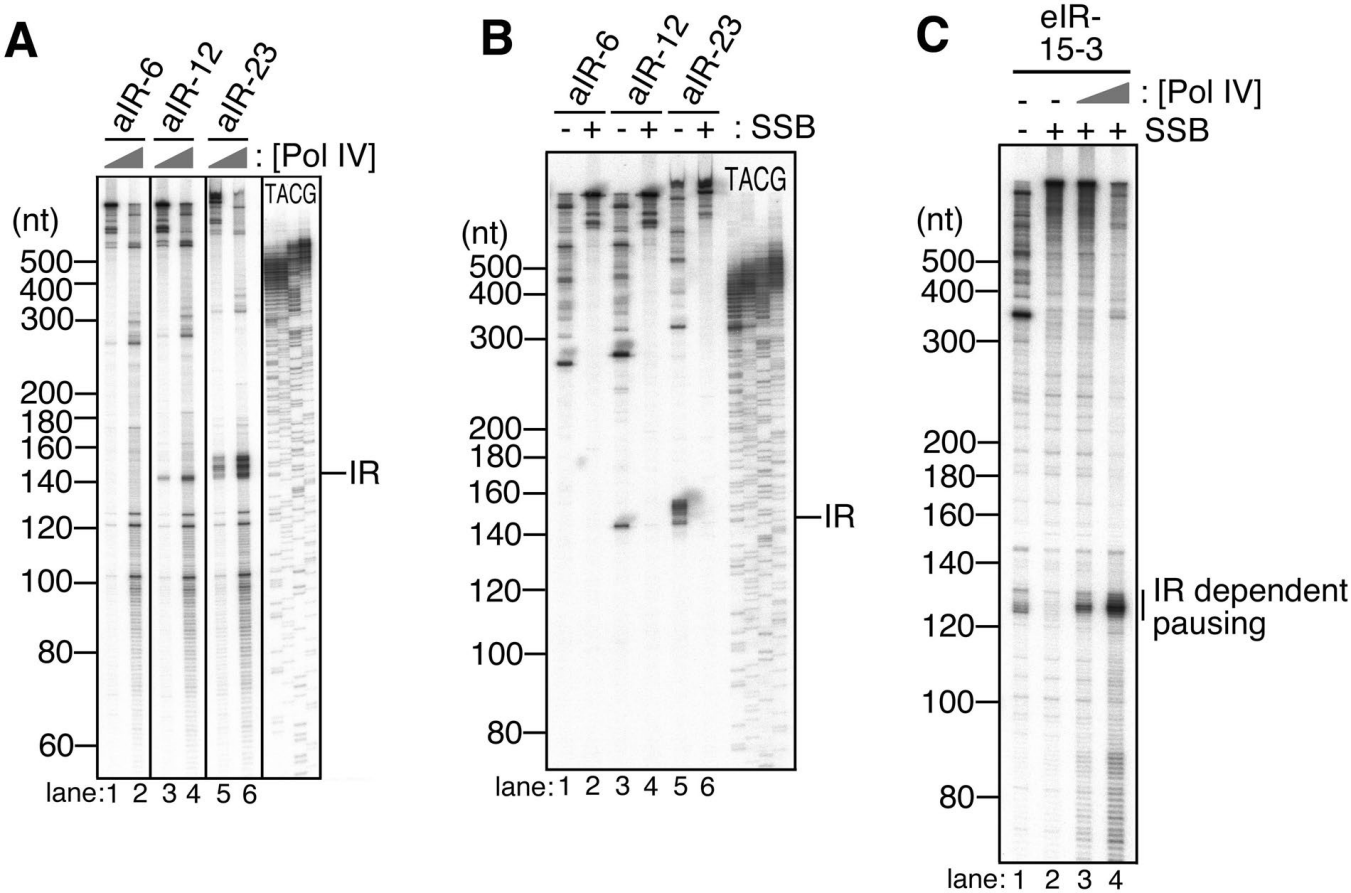
Pol IV-mediated Pol III pausing also occurs at inverted repeats with various length or sequence. (**A**) Template DNA carrying various sizes of inverted repeats, pMS2-aIR-6, -aIR-12 and -aIR-23 (6, 12, and 23 nt, respectively), were used for the assay to test the effect of Pol IV on Pol III. Wild-type Pol IV was added together with Pol III to the reaction at a final concentration of 10 and 100 nM (lanes 1–2, 3–4, and 5–6, respectively). After 3-minute pre-incubation, the elongation reactions were carried out for 10 seconds and replication products were analyzed as in Fig. 2B. 1-nt laddering markers representing sequences of pMS2-aIR-6 are shown on the right. (**B**) The behavior of Pol III HE in the presence or absence of SSB was analyzed on the DNA templates used in A. Laddering markers representing sequences of pMS2-aIR-12 are shown on the right. (**C**). The effect of Pol IV on Pol III was tested on a template carrying an *E. coli* endogenous inverted repeat (pMS2-eIR15-3) using wild-type Pol IV at a final concentration of 0, 10 and 100 nM as in A (lanes 2–4).

Our finding of Pol IV-dependent Pol III pausing at the repeat suggests that a hairpin structure exists on SSB-coated ssDNA and that Pol IV impedes Pol III-catalyzed elongation when Pol III encounters the hairpin structure. We also observed that Pol III pausing patterns caused by SSB omission and those caused by Pol IV are similar with different length and sequence of inverted repeats (Fig. 3). When pMS2-aIR-6 ssDNA carrying a 12-nt inverted repeat (a hairpin with 6-bp stem) was used as a template, Pol III neither paused at the repeat in the presence of Pol IV nor required SSB to replicate the repeat (Fig. 3A, lane 2, and 3B, lane 1). When pMS2-aIR12 carrying a 24-nt inverted repeat (a hairpin with 12-bp stem) was used, similar Pol IV- and SSB-omission-dependent pausing patterns were observed (Fig. 3A, lane 4, and 3B, lane 3). Additionally, the new template pMS2-eIR-15–3 carrying a 33-nt inverted repeat cloned from the *E. coli* genome (a hairpin with a 15-bp stem and 3-nt loop) gave us a similar result (Fig. 3C, compare lanes 1 and 4). These data suggest that a secondary structure, not a sequence, is the main cause of Pol IV-dependent Pol III pausing.

### Pol III-Pol IV switching is induced when Pol III collides with the hairpin, and the Pol IV- β complex replicates the hairpin-forming region

**Figure 4:**
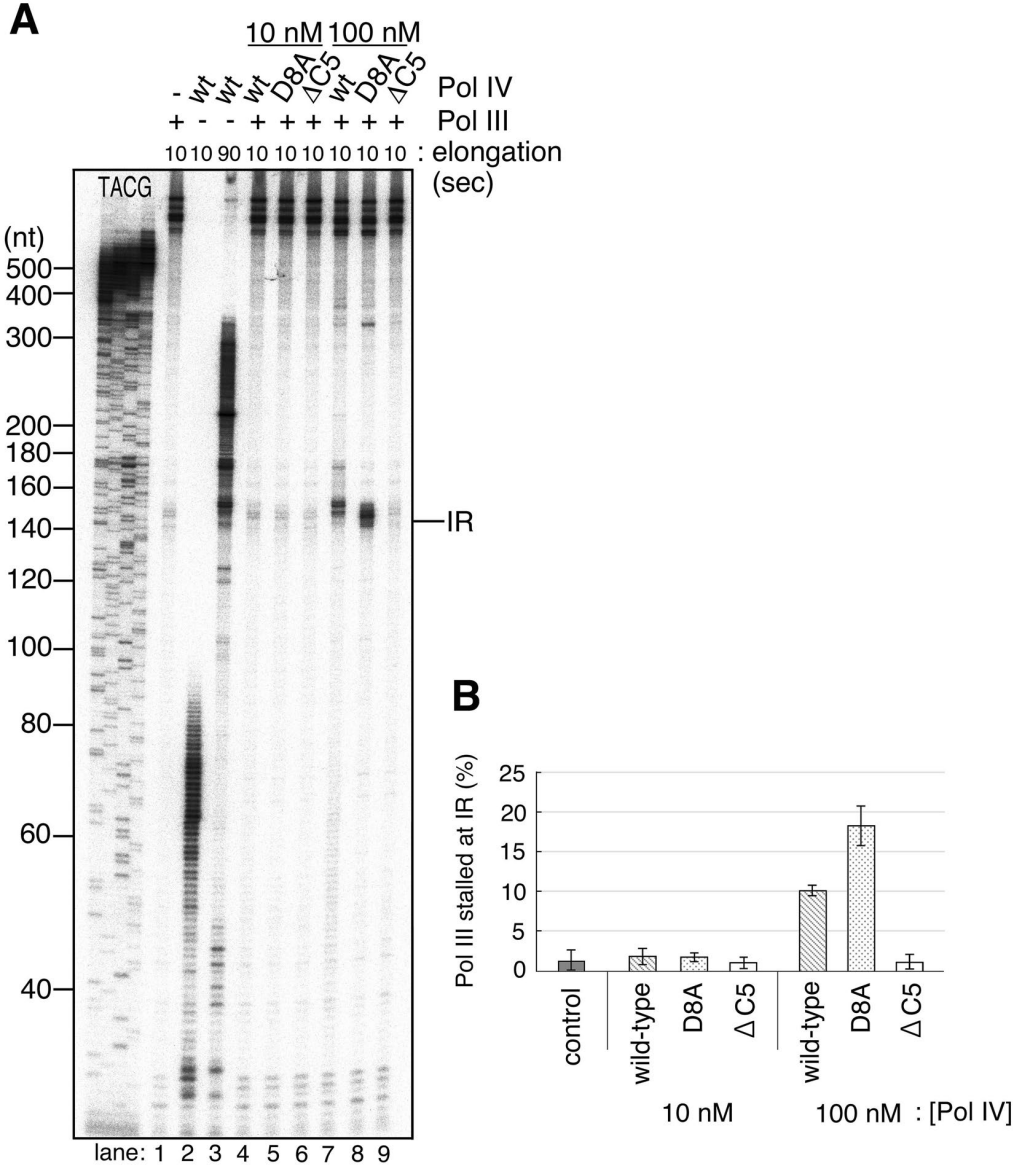
Pol III-Pol IV switch takes place when elongating Pol III collides with a hairpin structure on template ssDNA. (**A**) The effect of Pol IV on ongoing Pol III was tested on primed pMS2-aIR-23. Wild-type Pol IV or mutant Pol IV (D8A, ΔC5) was added to the reaction mixture (at a final concentration of 10 or 100 nM) together with dTTP when the elongation reaction started, and replication products at indicated time points were analyzed as in Fig. 2B. (**B**) The effect of wild-type or mutant Pol IV on ongoing Pol III at the repeat was quantitatively analyzed. The amount of pausing products at the repeat (between ~140 nt and ~191 nt) relative to the amount of total products in Fig. 4A were quantified and the percentages of stalled Pol III at the repeat were calculated. The average percentages of three independent experiments were shown with SD.

One possible explanation of Pol III pausing is that Pol IV simply inhibits the strand-displacement activity of Pol III. We reported that Pol IV has a physical interaction with SSB^29^, so it is possible that Pol IV competitively inhibits the interaction between Pol III and SSB and thus the strand-displacement activity, as PriA is reported to do^28^. However, it is unlikely because mutant Pol IV ΔC5, lacking the five C-terminal amino acids of the clamp-binding motif (CBM) failed to cause the pausing of Pol III at the repeat (Fig. 4A, compare lanes 7 and 9). Pol IV ΔC5 is predicted to bind to SSB, as Pol IV lacking its C-terminal one third still binds to SSB^29^. The other possibility that Pol IV itself binds to the hairpin to cause Pol III stalling is also unlikely because Pol IV is reported to preferentially bind to ssDNA^30^ and Pol IV ΔC5 can bind ssDNA as well as wild-type Pol III does (Supplemental Figure S2).

More likely is that the polymerase switch from Pol III to Pol IV takes place when Pol III encounters a hairpin. If Pol III-Pol IV switching occurs, the pausing products should appear because of the slower elongation speed or lesser strand-displacement activity of Pol IV. This idea is strongly supported by the fact that Pol III pausing was not observed when mutant Pol IV ΔC5 was used (Fig. 4A, lane 9), as the clamp binding is essential for Pol IV to switch places with stalled Pol III at the primer terminus^20,29,31,32^.

**Figure 5:**
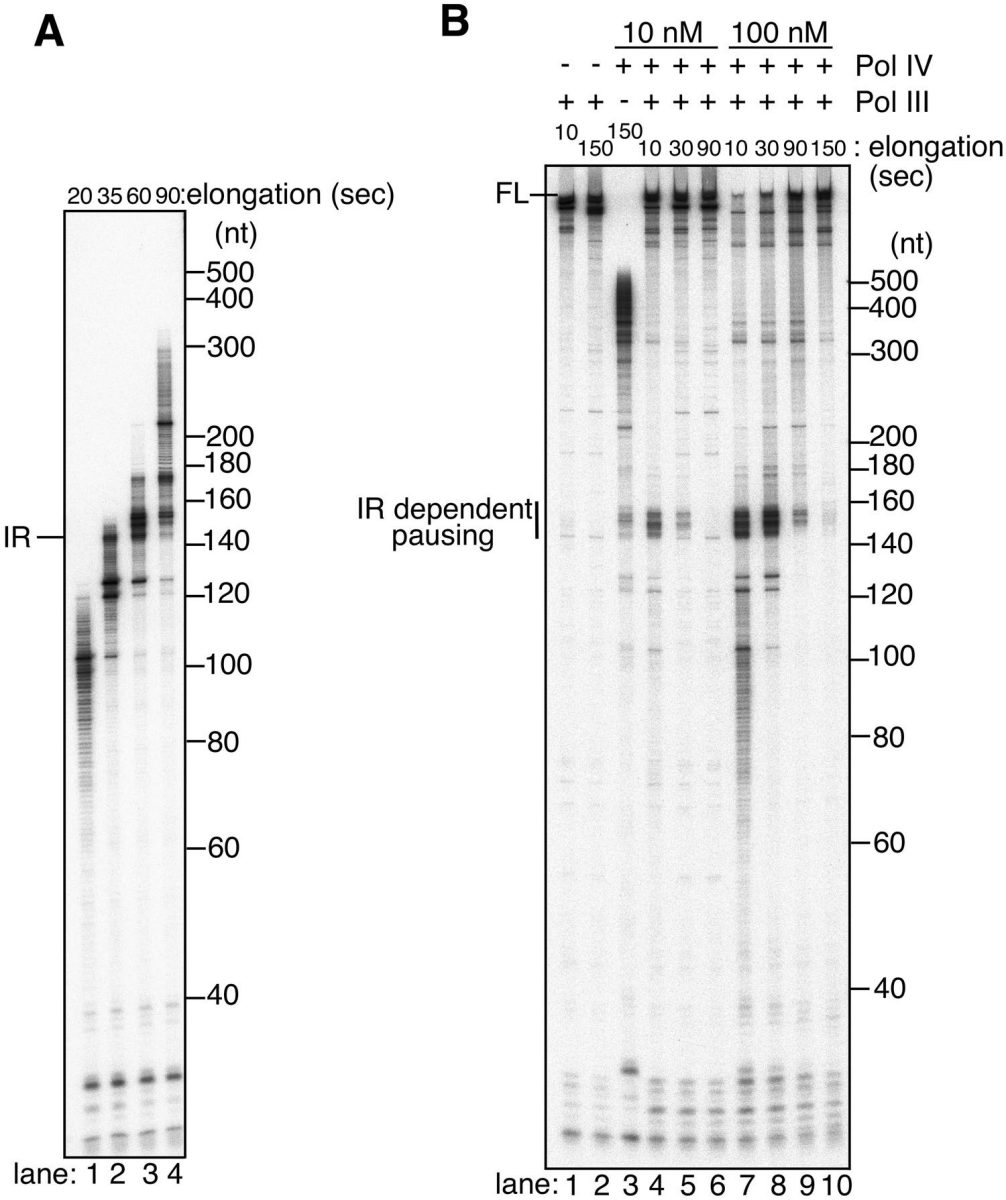
Two sequential polymerase switches rapidly take place to replicate across the hairpin structure by Pol III and Pol IV. (**A**) The behavior of Pol IV-β complex was analyzed on primed pMS2-aIR-23. The γ complex loads the β clamp onto template DNA instead of Pol III. Pol IV bound to the β clamp solely elongates the primer for indicated time. See Materials and Methods for details. (**B**) The effect of Pol IV on Pol III was tested on primed pMS2-aIR-23 in the time course experiment. Wild-type Pol IV was added at a final concentration of 10 nM (lanes 4–6) or 100 nM (lanes 7–10) together with Pol III. Replication products at indicated time points were analyzed as in Fig. 2B. The amount of pausing and full-length products in Fig. 5B were quantified and the percentages of each product were shown in Supplemental Figure S3A. The quantitative analysis was independently repeated and the averaged data was shown in Supplemental Figure S3B.

In Fig. 2B, short products (<120 nt) formed by the switch from stalled Pol III to Pol IV were observed for both templates because Pol IV was added during the first pre-incubation. In the experiment in Fig. 4, wild type- or mutant Pol IV was added together with dTTP when Pol III-catalyzed burst DNA synthesis was started to test the effect of Pol IV on ongoing Pol III HE. Thus, Pol III initially extended all of the primer and the short Pol IV-catalyzed products were not observed in this experiment. Pausing products were difficult to be detected at 10 nM Pol IV, but they clearly appeared when 100 nM wild-type Pol IV was used within long products extended by Pol III, showing that Pol IV inhibits the elongating Pol III HE at the hairpin site (Fig. 4A, lane 7). The pausing signals with wild-type Pol IV located around the middle of the upstream half of the repeat, while those in Fig. 2 were found from the beginning to the middle of the repeat. An activity-deficient mutant Pol IV, D8A, gave even stronger pausing signals than wild-type Pol IV, while the CBM-deficient mutant Pol IV ΔC5 failed to produce the pausing signals at the hairpin (Fig. 4A, compare lanes 7, 8 and 9). Notably, pausing products of wild-type Pol IV were slightly longer than those of Pol IV D8A, showing that the polymerase activity of Pol IV participates in the extension of the primer at the repeat. Taken together, we conclude that the appearance of the pausing bands is caused by the polymerase switch from Pol III to Pol IV. Because the pausing site with D8A starts at the beginning of the repeat, we speculate that the switch occurs just after Pol III collides with the hairpin. When the burst synthesis is initiated, Pol III quickly extends the primer until the repeat within a short time (~0.15 sec, 900 nt/sec). Then Pol IV takes over the CBM-binding pocket of the β clamp and the primer terminus from Pol III. Pausing signals caused by wild-type Pol IV were 5–10 nt inside the repeat, probably because Pol IV elongates the primer while unwinding the hairpin during the rest of incubation time by its strand-displacement activity (~1 nt/sec, Fig. 5A).

### The second polymerase switch, from Pol IV to Pol III, takes place after Pol IV overcomes the hairpin

When Pol IV alone replicates DNA with the β clamp, Pol IV is able to elongate the primer across the hairpin, although its elongation speed there becomes approximately one-fifth of the normal speed (normal: ~5 nt/sec, hairpin: ~1 nt/sec, Fig. 5A). Its product lengths are shorter than 80-nt within 10 seconds and reached ~300 nt after 90-second incubation (Fig. 5A, lane 4). On the other hand, when Pol III was incubated together with 100 nM Pol IV, the pausing products (~145–150 nt) appeared within 10 seconds and gradually decreased over the time while the full-length products increased (Fig. 5B, lanes 7–10, Supplemental Figure S3A, right panel). After a 90-second incubation, a significant amount of long, near-full-length and full-length products were produced (Fig. 5B, lane 9). Considering the slow elongation speed of Pol IV and the fact that characteristic 1-nt laddering patterns of Pol IV products were not observed downstream of the hairpin, we concluded that soon after the hairpin-bypass by Pol IV, a second polymerase switch (from Pol IV to Pol III) takes place and produces the full-length products.

### The interaction between Pol III HE and template DNA may modulate Pol IV-mediated polymerase switching

In cells, the polymerase switch is believed to occur for TLS when Pol III is blocked by a lesion. However, the switch observed in this work is presumably between ongoing Pol III and Pol IV because the idea that Pol III stalls at such a short hairpin in the presence of SSB is not evident. It has been reported that Pol III, unlike other replicative polymerases, continues the primer elongation when it collides with 5′-flapped duplex DNA on an ssDNA template and that this continued elongation takes place with robust strand-displacement activity at a speed of ~150 nt/sec and with the processivity of ~280 nt^28^. Thus, even if Pol III encounters the short hairpin on our ssDNA template, it should overcome the 23-bp duplex stem within 0.15 seconds without any dissociation because the structure of the hairpin is similar to that of a 5′-flapped duplex. Supporting this idea, we observed that Pol III smoothly replicated the inverted repeat without any significant pausing even if approximately 1000-fold molar excess of mutant Pol IV *231–351* (the C-terminal one-third of Pol IV that contains the entire little finger domain with C-terminal CBM) over Pol III were added to the reaction (Supplemental Figure S4C, lane 3). Because the pull-down assay shows that Pol IV *231–351* stably binds to the β clamp as wild-type Pol IV does (Supplemental Figure S4B, compare lanes 1 and 3), those data further indicates that the polymerase switch observed here is not caused by a spontaneous Pol III dissociation and following a simple competition for binding sites on the β clamp.

On the other hand, we cannot deny the possibility that Pol III transiently pauses just for much less than one second at the hairpin site, as faint pausing bands appeared even in the absence of Pol IV (Fig. 2B, lane 6), but it is unclear how Pol IV efficiently takes over the β clamp/primer terminus within such a short period. Elongating Pol III HE is tightly anchored to the primer/template junction by the β clamp. It is also thought to be in contact with template ssDNA via multiple weak ssDNA binding motifs in the core polymerase (α-ε-θ) and the DnaX clamp loader complex. Interestingly, it has been reported that DNA binding induces a large conformational change of the Pol III core on the β clamp^33^. Thus, we hypothesized that when one of these interactions with template DNA is lost because of the presence of duplex ahead of the enzyme, even if other interactions still anchor Pol III HE to DNA tightly enough to continue the strand-displacement synthesis, the loss of an interaction may induce a conformational change of Pol III HE and weaken the interactions between the Pol III core and the β clamp, which may allow Pol IV to take over the CBM-binding pocket and the primer terminus.

**Figure 6:**
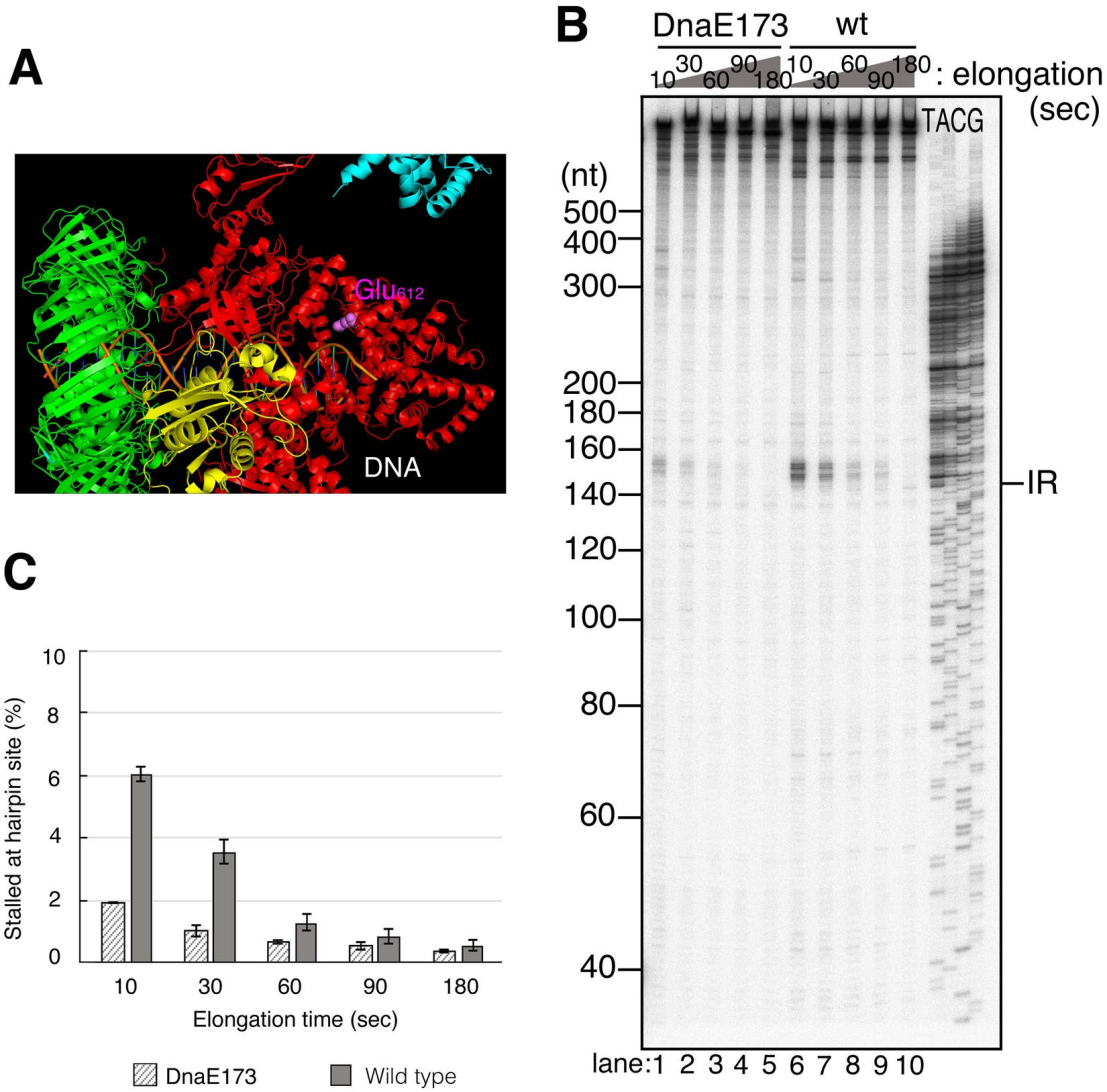
Pol III_*dnaE173*_ HE is more resistant than wild-type Pol III HE to Pol IV-mediated switching at the inverted repeat. (**A**) Structural model of Pol III α-ε on β-DNA complex showing the location of a mutation in *dnaE173* (based on PDB 5fkv^33^, and created using MacPyMOL (DeLano Scientific LLC)). Glu^612^ is shown as pink spheres. Subunits are shown as colored ribbons: α, red; ε, yellow; β, green: τ (partial), pale blue. (**B**) The effect of Pol IV on ongoing wild-type or mutant Pol III HE was tested on primed pMS2-aIR-23 in the time course experiment. Wild-type Pol IV was added to the reaction at a final concentration of 100 nM when the elongation reaction was initiated by the addition of dTTP. Replication products at indicated time points were analyzed as in Fig. 2B. (**C**) The amount of pausing products at the repeat relative to the total products each conditions in Fig. 6B were quantified. The percentages of stalled Pol III in the presence of 100 nM Pol IV at the repeat were calculated and the average of three independent experiments were shown with standard deviations (SD).

To test this possibility, we used a mutant Pol III with an increased affinity for template ssDNA, Pol III_*dnaE173*_ HE^25^. It has an amino acid substitution of negatively charged Glu by positively charged Lys at residue 612 of the finger domain of the α subunit^34^. The mutation is predicted from cryo-EM structure analysis to be on a protein surface where the enzyme is in closely contact with DNA^33^ (Fig. 6A). A previous analysis had revealed that the α subunit with this mutation shows a stronger affinity to primed ssDNA than the wild-type shows^35^. Pol III_*dnaE173*_ HE exhibits various characteristic behaviors including a slow elongation speed and elevated processivity and strand-displacement activity^25^. Its most striking characteristic is a severe proof-reading deficiency evident even when the holoenzyme has the fully active ε subunit^34^. The rate of spontaneous mutation in *dnaE173* cells is about 10,000-fold greater than that in wild-type cells. This suggests that the conformational change of Pol III core required for the α-ε exchange is inhibited by this mutation because of the high affinity of the α to ssDNA. In addition, we observed that most Pol III_*dnaE173*_ HE overcomes the hairpin even in the absence of SSB (Supplemental Figure S5, compare lanes 5 and 10). This also suggests that Pol III_*dnaE173*_ HE keeps moving while unwinding a duplex by itself and cannot shift the mode suitable for the canonical SSB-dependent strand displacement. Probably the strong affinity of the α subunit to ssDNA fixes the Pol III_*dnaE173*_ core on the β clamp in the normal elongation mode and does not allow it to change the mode even if it incorporates a wrong dNTP or encounters a duplex. As predicted, Pol III_*dnaE173*_ HE becomes more resistant to Pol IV than wild-type Pol III (Fig. 6B and C). Pol IV-dependent Pol III_*dnaE173*_ HE pausing signals at the hairpin were faint and decreased faster than those of wild-type Pol III HE (compare lanes 1–5 and 6–10 in Fig. 6B). We speculate that most Pol III_*dnaE173*_ HE does not change the mode even if it collides with a secondary structure so that there is lesser chance for Pol IV to take over the CBM-binding pocket on the clamp.

Previously, we observed that Pol III HE detached from the clamp when Pol IV takes over the primer and the clamp from stalled Pol III^21^. It is possible that Pol III dissociation is also induced by the effect of Pol IV at the hairpin. If the Pol III core detaches from the clamp, the Pol III_*dnaE173*_ results may suggest that the increased affinity to ssDNA may help Pol III_*dnaE173*_ core regain access to the primer/template junction from Pol IV to restart Pol III-catalyzed primer extension. Currently, it is unclear whether the Pol III core remains attached to the clamp and template DNA during the process of polymerase switching in our system.

### Pol IV takes over the β clamp and the primer terminus from Pol III when Pol III HE collides with a downstream primer/template duplex

**Figure 7:**
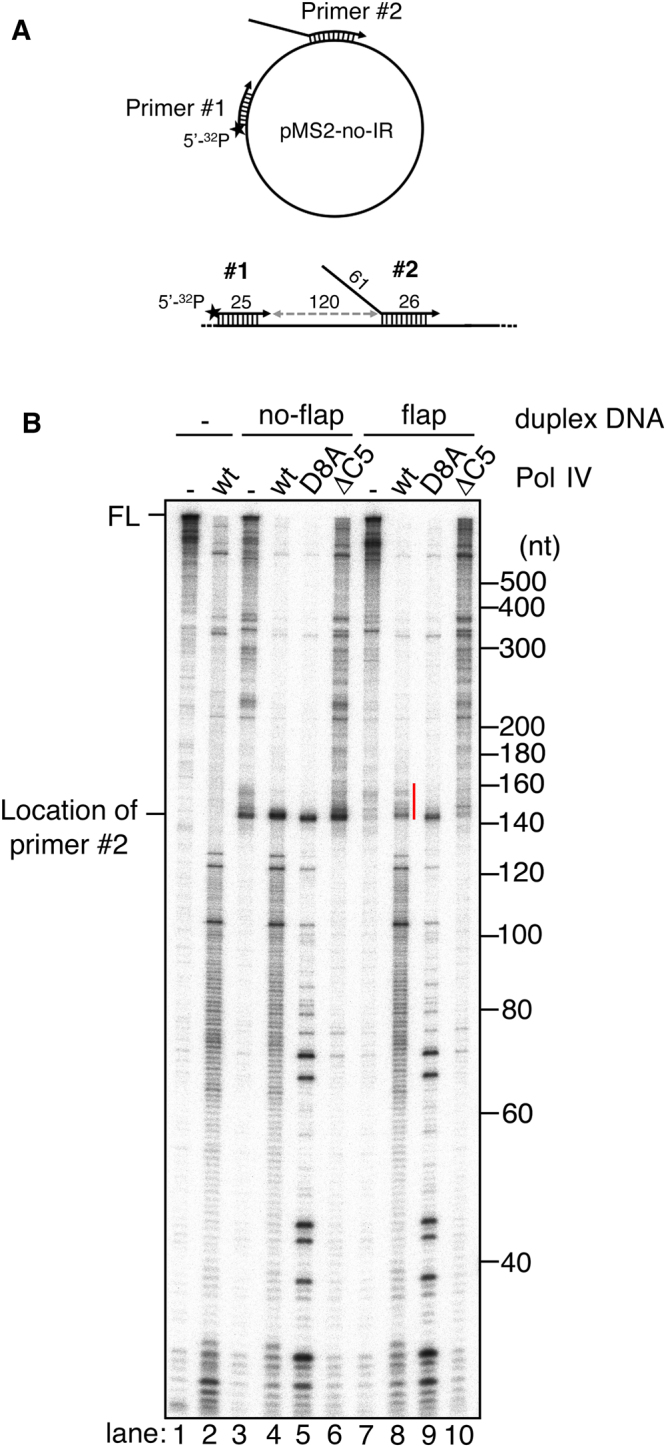
Pol III-Pol IV switching also occurs when Pol III collides with a flapped duplex on template DNA. (**A**) *upper*: Template DNA used in this assay. A circular ssDNA without an inverted repeat was annealed with 5′-^32^P labeled primer (primer #1) and 61-nt flapped primer (primer #2). The location of a radiolabel is indicated as a black star. *lower:* Sketch showing the location of two primers. (**B**) The effect of Pol IV on wild-type Pol III HE was tested on the double-primed template. Note that the amount of Pol III* used in this assay was double those used in the other experiments. Template DNA shown in A was used in lanes 7–10. A singly primed template (with primer #1 only, lanes 1 and 2) and no-flap template (with primers #1 and #2 but without 61-nt flap, lanes 3–6) were used as controls. Wild-type or mutant Pol IV (D8A or ΔC5) was added to the reaction together with Pol III at a final concentration of 0 or 100 nM as indicated. Replication products after 10-second elongation reaction were analyzed as in Fig. 2B.

If the above hypothesis is correct, not only a hairpin but also any other type of duplex on a template should make Pol III HE susceptible to Pol III-Pol IV switching. To test this possibility, we analyzed the behavior of Pol III HE when it collides with the 5′-end of the primer on template DNA by using a circular ssDNA template annealed with two primers, one ^32^P-labeled and the other not (respectively primers #1 and #2 in Fig. 7A). In this assay, two Pol III HE forms initiation complexes on both primers and simultaneously starts elongations. Pol III HE elongating the ^32^P-labeled primer #1 collides with the 5′-end of the primer #2 after replicating a short distance (120 nt). Because the interaction between Pol III HE and SSB is required for the strand displacement reaction^28^, when no-flapped primer was used as primer #2, approximately half the Pol III HE stopped the elongation when it collided with primer #2, while the rest continued the elongation by strand displacement activity (Fig. 7B, lane 3). On the other hand, when a 61-nt ssDNA flap was present at the 5′-end of primer #2, Pol III HE robustly continued the strand-displacement-type elongation beyond the second primer site with the aid of SSB and produced near-full-length products within the incubation time (Fig. 7B, lane 7).

When wild-type Pol IV was added to the reaction together with Pol III, short, 1-nt laddering products with the size of <120–130 nt appeared independently of the presence of the second primer as a result of the polymerase switch from Pol III to Pol IV during the pre-incubation (Fig. 7B, lanes 2, 4 and 8). Addition of Pol IV D8A resulted in the appearance of several intense pausing signals instead of the 1-nt laddering (Fig. 7B, lanes 5 and 9). This result implies that Pol III-Pol IV switch occurs prior to Pol III encountering the second primer, probably caused by naturally-formed secondary structures on ssDNA template with an ordinary sequence.

As expected, the addition of wild-type or Pol IV D8A caused the appearance of pausing bands at the starting position of primer #2 (~145 nt, compare lane 7 with lanes 8 and 9 in Fig. 7B). Remarkably, the further elongation of primer #1 by Pol III HE across this site was largely shut down and neither full-length nor near-full-length products were observed when wild-type Pol IV or Pol IV D8A was added (Fig. 7B, lanes 4, 5, 8, and 9). The extension of paused products with lengths up to ~20–30 nt was seen for wild-type Pol IV at flapped primer #2 (shown as a red line in the image of Fig. 7B, lane 8) but not for Pol IV D8A (Fig. 7B, lane 9). In contrast, Pol III HE could produce a significant amount of full- or near-full length products by the strand-displacement activity even in the presence of Pol IV ΔC5 (Fig. 7B, lane 10), although weak inhibitory effects were observed probably via the inhibition of Pol III-SSB interaction.

These data suggest that Pol III-Pol IV switching also occurs when Pol III HE collides with the 5′-end of primer #2 and Pol IV rather than Pol III HE elongates the primer #1 by strand displacement activity with the aid of SSB on the flap, as observed at the hairpin-containing template. Notably, in Fig. 5B it seems that Pol III quickly takes the primer end back from Pol IV downstream of the short hairpin, but it seems in Fig. 7 that Pol III is difficult to return to the primer end during the incubation. These data suggest that as long as a duplex exists and if Pol III HE cannot restore the interaction with ssDNA, Pol IV dominates the primer end and prevents the strand-displacement type elongation by Pol III HE.

## Discussion

Here we biochemically studied how the polymerase switch from Pol III to Pol IV is initiated when Pol III HE is elongating the primer end for better understanding of various cellular functions of Pol IV. We observed that a short hairpin or a duplex on a template strand effectively induces switching from Pol III to Pol IV in a lesion-independent manner. Pol III HE becomes capable of Pol IV-mediated switching when it collides with the duplex stem of a hairpin or the 5′-end of a primer/template and starts strand displacement synthesis. Our data suggest that the loss of Pol III interaction with ssDNA, presumably the α-ssDNA interaction, may trigger the handover from Pol III to Pol IV. Interestingly, in previous reports using a single-molecule assay to analyze the dynamics of polymerase exchange between Pol III and Pol IV, Pol IV-mediated Pol III dissociation from the clamp was suggested^14,36^. Because long SSB-free ssDNA was used as a substrate in the single-molecule assay, it is also possible that the observed polymerase exchanges were triggered by a secondary structure on ssDNA^14,36^.

Why does switching from Pol III to Pol IV take place when Pol III collides with a duplex and loses contact with ssDNA? A previous report proposed an idea that the α-ssDNA interaction controls Pol III releasing from the clamp on the lagging strand synthesis. In this ‘collision model’, it is hypothesized that the OB fold in the α subunit act as a sensor stimulating the core recycling when it collides with a 5′-end of the primer for Okazaki fragment synthesis^37^. In our system, however, it is highly unlikely that Pol III spontaneously dissociates from the clamp at the hairpin and offers Pol IV a chance to take over the clamp, since Pol III HE continues a robust strand displacement synthesis even if it encounters a duplex because 5′-flap ssDNA-SSB-Pol III (DnaX(τ-ψ-χ)-core) interaction tethers Pol III HE to the primer/template junction^28^. It is also reported that Pol III* stays on the clamp for ~2 min after an ssDNA gap becomes a nick^38^, and a ‘signaling model’ in which the presence of primer and the action of DnaX complex plays an important role in polymerase recycling has been proposed^39,40^. In addition, mutant Pol IV *231–351* does not competitively inhibit elongating Pol III at the hairpin, even if it can stably bind to the β clamp via its C-terminal CBM (Supplemental Fig. S4B and 4C). Our data suggest that Pol III does not spontaneously dissociate from the clamp and the primer at the hairpin, and if the Pol III does, that should be a consequence, not a cause, of Pol IV-mediated switching. As previously we and others hypothesized, Pol IV may have an as-yet-unidentified interaction with Pol III HE to stimulate Pol III dissociation from the clamp in its N-terminal portion, as the binding of C-terminal little finger domain (Pol IV *231–351*) to the clamp is not sufficient to cause the polymerase switch^21,32^.

Another possible explanation is that switching from Pol III to Pol IV is triggered by an overall interactional/structural change of Pol III HE caused by collision with a duplex. Our data suggest that the loss of Pol III-ssDNA interaction may allow Pol IV to take over the clamp-primer/template junction from Pol III. When Pol III HE normally elongates a primer, the α subunit on the β clamp primarily holds the primer end while contacting a template ssDNA strand. The ε subunit simultaneously binds to the β clamp and accesses the primer end when it excises a misincorporated nucleotide. The DnaX complex also interacts with the Pol III core, the clamp, template ssDNA, and SSB and participates in the processive elongation. These multiple interactions change when Pol III HE collides with a duplex and starts invading it. Pol III core-DnaX(τ-ψ-χ)-SSB on the 5′-flap becomes critical during the strand displacement synthesis as mentioned earlier^28^. Interestingly, the ε subunit also becomes more important for the primer extension in the strand displacement mode than in the normal elongation mode^41^. We speculate that the weakened affinity of the α subunit to template ssDNA induces the exchange of an enzyme at the primer end between the α and the ε subunits when the ssDNA region disappears, and perhaps this exchange may give a chance for Pol IV to access the primer end and the CBM-pocket. Supporting this idea, it has been reported that a duplex ahead of the enzyme induces viral and eukaryotic replicative Pols in the exonuclease mode and the loss of exonuclease activity of Pol ε strongly stimulates strand-displacement activity^42,43^. Perhaps, duplex-induced frequent change of modes between polymerization and exonuclease activity is conserved in Pols of many organisms.

If the above hypothesis is correct, it is possible that an initial step in the process of Pol III-Pol IV switching may be the same at a duplex observed here and at a lesion during TLS. When Pol III HE encounters an obstacle, it does not simply stall but repetitively changes modes between polymerase and exonuclease. In this ‘idling’ state the subunits at the primer end repeatedly switch between the α and ε subunits on the clamp. Thus, as hypothesized above, if the exchange of subunits at the primer end caused by the loss of interaction with ssDNA triggers the handover from Pol III to Pol IV, ‘idling’ Pol III would for the same reason easily allow Pol IV to take over the primer terminus.

In our *in vitro* system, we observed that Pol IV primarily synthesizes DNA containing hairpin-forming, short-inverted repeat even though its catalytic activity is poorer than that of Pol III. In addition, our data imply that Pol IV-mediated polymerase switch from Pol III to Pol IV can be initiated by a weak secondary structure formed at non-specific sequences (Fig. 7B, lanes 5 and 9). It is interesting whether Pol IV participates in the replication of hairpin and other types of non-B structures in *Escherichia coli* cells. The prokaryotic genome contains fewer such sequences than the eukaryotic genome does, but short inverted repeats less than ~30 bp long are relatively abundant in it^44^. Interestingly, a mutation at a hotspot inside an imperfect palindrome in *thyA* gene is suppressed by the presence of Pol II, IV or V, implying that Pol IV participates in the replication of a hairpin formed in the repeat^45^. In addition, Pol IV-dependent mutation is observed on the lagging strand a little more frequently than on the leading strand^46^. It is possible that Pol IV-mediated switching may occur at a secondary structure formed within ssDNA regions during Okazaki fragment synthesis. On the other hand, it is possible that Pol III on the leading strand may encounter a secondary structure within a duplex in front of the fork, as DnaB helicase is sliding on the lagging strand. Pol III-Pol IV switching on the leading strand may slow replication as observed *in vivo*. It has been reported that eukaryotic translesion Pols can participate in replication of non-B DNA when replicative Pol δ stalls at such sequences and contributes to increasing the mutation frequency^47^. Although the molecular mechanisms seems to be different, as the arresting of replicative Pol activity is not required for *Escherichia coli* Pol III-Pol IV switching, maybe translesion Pols may have conserved roles for replication of non-B DNA. Further investigation is needed to find out whether Pol IV also contributes to error-prone/-free replication at non-B sequences. One interesting observation is that Pol III HE is difficult to replicate a flapped duplex DNA when Pol IV is present, even though the strand-displacement activity of Pol III HE is much stronger than that of Pol IV (Fig. 7B). Previous *in vivo* and *in vitro* data suggest that Pol IV is recruited at displacement loops (D-loops) and participates in error-prone recombination at a dsDNA break site in stress-induced cells^11–13^. Our data may explain the handover of the β clamp from Pol III to Pol IV at D-loops and how Pol IV exclusively extends the primer during error-prone recombination in cells.

## Materials and Methods

### Proteins

SSB, wild-type or *dnaE173* Pol III* was purified with CBP-tag on ψ subunit as previously described^48^. The γ complex was a generous gift from Dr T. Katayama (Kyushu University). The β clamp was prepared as described^25^. His-tagged wild-type or mutant Pol IV (D8A, ΔC5, *1-230* and *231–351*) were purified as previously described^21^ (purified proteins are shown in Supplemental Figure S4A).

### DNA template

The inverted repeat phagemid pMS2 was the generous gift from Dr M. Moriya (Stony Brook University). To distinguish original pMS2 carrying 46-bp inverted repeat from other phagemids we constructed, we call pMS2 as pMS2-aIR-23 in this work. Other phagemids carrying shorter inverted repeats pMS2-aIR-12 (12 nucleotides in each arm) and pMS2-aIR-6 (6 nucleotides in each arm) were prepared by digestion of pMS2-aIR-23 with *Sal*I or *Eco*RV, which are located symmetrically in both arms of the inverted repeat, respectively, followed by ligation and *E. coli* transformation. A control phagemid carrying no inverted repeat (pMS2-no-IR) was prepared from single-stranded pMS2-aIR-23 by inserting an unrelated 60-nt sequence between *EcoR*V sites as previously described^27^. To prepare a pMS2-based phagemid containing an *E. coli* endogenous inverted repeat, an inverted repeat that consists of 15 nucleotides in each arm linked by 3-nucleotides spacers (5′ AAA TGG ACG GCG ATG TAT CAT CGC CGT CCA TTT 3′) was amplified from MG1655 genome by PCR (forward primer: 5′ TGC AGG TAC CTT AGA TTA TCC TGA TTA TAA ACG 3′; reverse primer: 5′ GAC TGG GCC CTG ATG CGT TCC TCA CTT G 3′) and inserted into pMS2-no-IR phagemid by *Kpn*I - *Apa*I site. Circular ssDNA templates for DNA synthesis assay were prepared from each phagemid as previously described^49^ except that the CsCl purification step was omitted.

### Preparation of primed-ssDNA templates

Primers named as pri3000 (5′ GAG ATA GGG TTG AGT GTT GTT CCA GT 3′); pri145 (5′ AGA GCA GCC GAT TGT CTG TTG TGC C 3′) and pri125 (5′ AAT CAT GCG AAA CGA TCC TCA TCC TG 3′) were ^32^P-labeled at the 5′ end by using T4 kinase (Toyobo). To prepare singly-primed ssDNA template, an appropriate 5′-^32^P labeled primer was annealed with circular ssDNA template (pri3000/pMS2-aIR-23 for alkaline agarose gel electrophoresis analysis in Fig. 1D; pri145/pMS2-noIR, -aIR-23, -aIR-12, -aIR-6, and pri125/pMS2-eIR-15-3 for sequencing gel electrophoresis analysis in Figs 2–7) at a molecular ratio of 3:1 followed the purification to remove free primer by Microspin S-400HR (GE healthcare). To prepare a double-primed ssDNA template, 5′-^32^P labeled pri145 and non-labeled primer H (5′ AG CGA TTG CAT AAG CTT TTG CCA TTC 3′) or non-labeled primer H + 61-nt flap (5′ *GCT TTG CCA CGG AAC GGT CTG CGT TGT CGG GAA GAT GCG TGA TCT GAT CCT TCA ACT CAG C-*AG CGA TTG CAT AAG CTT TTG CCA TTC 3′, 61-nt flap sequence is shown in italics) were annealed with ssDNA pMS2-no-IR and purified as described earlier.

### Two-step DNA synthesis assay (burst DNA synthesis assay) with Pol III and Pol IV

For experiments in Figs 1–6, singly primed ssDNA template (0.03 pmol), SSB (0.6 μg), the β clamp (0.12 μg) and wild-type or mutant Pol III* (20 units) were pre-incubated in the first reaction mixture (7.5 μl) containing EDBG (20 mM Tris-HCl (pH 7.5), 4% glycerol, 8 mM dithiothreitol, 80 μg/ml bovine serum albumin), 1 mM ATP, 8 mM MgCl_2_ and 100 μM each of dATP, dCTP, and dGTP at 30 °C for 3 min to form the initiation complex. Primer elongation reaction was initiated by adding 100 μM dATP, dGTP, and dCTP and 133 μM dTTP to pre-warmed second reaction mixture (22.5 μl) containing EDBG, 1 mM ATP and 8 mM MgCl_2_. The final concentrations of primed DNA and Pol III are estimated as 1 nM and 1.2 nM in the reaction mixture (30 μl), respectively^21^. After the reaction was incubated at 30 °C for the indicated time in each Figure legend, the reaction was terminated by the addition of 140 μl of stop solution (50 mM EDTA, 0.15% SDS, pH 8.0). Wild-type or mutant Pol IV was added to the first or second reaction mixture at indicated concentrations and time points as described in each Figure legend. Replication products were purified and analyzed by either alkaline 0.9% agarose gel electrophoresis^21^ or 8M-Urea 7.5% polyacrylamide denaturing sequencing gel electrophoresis, followed by autoradiography. The results were analyzed using BAS-2500 Phosphor Image Scanner (Fujifilm) and Multi Gauge Software (Fujifilm). Quantifications were carried out using photostimulated luminescence value of radioactive signals of the gel images. In Fig. 7, assays were carried out as in Figs 1–6 except that double-primed ssDNA template (0.03 pmol), SSB (0.6 μg), the β clamp (0.24 μg), and wild-type Pol III* (40 units) were used to form two initiation complexes on one template DNA molecule.

### Two-step DNA synthesis assay with Pol IV

The first reaction mixture (7.5 μl) was prepared as described earlier except that the γ complex (5 nM) was added instead of Pol III*. The final concentration of 100 nM Pol IV was also included in the reaction. During the first pre-incubation, the γ complex loads the β clamp onto SSB-coated primed ssDNA template. The replication products were produced and analyzed as described earlier.

## Electronic supplementary material

Supplemental Information

## References

[1] Kornberg A., Baker T. *DNA replication, second edition*. (W.H. Freeman and Company, New York, 1992).

[2] Yao N, O’Donnell M (2016). Bacterial and Eukaryotic Replisome Machines. JSM Biochem. Mol. Biol..

[3] Maki H., and Furukohri A. in DNA Polymerase III, Bacterial. In: Lennarz W.J. and Lane M.D. (eds.) The Encyclopedia of Biological Chemistry, vol. 2, pp. 92–95. (Academic Press, Waltham, MA, 2013).

[4] McHenry CS (2011). Bacterial replicases and related polymerases. Curr. Opin. Chem. Biol..

[5] Butland G (2005). Interaction network containing conserved and essential protein complexes in *Escherichia coli*. Nature.

[6] Friedberg E.C., *et al*. In *DNA repair and mutagenesis. 2nd ed*. (ASM Press, Washington DC, 2006).

[7] Fuchs RP, Fujii S (2013). Translesion DNA synthesis and mutagenesis in prokaryotes. Cold Spring Harb Perspect. Biol..

[8] Shen X (2002). Efficiency and accuracy of SOS-induced DNA polymerases replicating benzo[a]pyrene-7,8-diol 9,10-epoxide A and G adducts. J. Biol. Chem..

[9] Jarosz DF, Godoy VG, Delaney JC, Essigmann JM, Walker GC (2006). A single amino acid governs enhanced activity of DinB DNA polymerases on damaged templates. Nature.

[10] Bjedov I (2007). Involvement of *Escherichia coli* DNA polymerase IV in tolerance of cytotoxic alkylating DNA lesions *in vivo*. Genetics.

[11] Ponder RG, Fonville NC, Rosenberg SM (2005). A switch from high-fidelity to error-prone DNA double-strand break repair underlies stress-induced mutation. Mol. Cell.

[12] Mallik S, Popodi EM, Hanson AJ, Foster PL (2015). Interactions and Localization of *Escherichia coli* Error-Prone DNA Polymerase IV after DNA Damage. J. Bacteriol..

[13] Pomerantz RT, Kurth I, Goodman MF, O’Donnell ME (2013). Preferential D-loop extension by a translesion DNA polymerase underlies error-prone recombination. Nat. Struct. Mol. Biol..

[14] Scotland MK (2015). A Genetic Selection for dinB Mutants Reveals an Interaction between DNA Polymerase IV and the Replicative Polymerase That Is Required for Translesion Synthesis. PLoS Genet..

[15] Sutton MD (2010). Coordinating DNA polymerase traffic during high and low fidelity synthesis. Biochim. Biophys. Acta.

[16] Kim SR (1997). Multiple pathways for SOS-induced mutagenesis in *Escherichia coli*: an overexpression of dinB/dinP results in strongly enhancing mutagenesis in the absence of any exogenous treatment to damage DNA. Proc. Natl. Acad. Sci. USA.

[17] Wagner J, Nohmi T (2000). *Escherichia coli* DNA polymerase IV mutator activity: genetic requirements and mutational specificity. J. Bacteriol..

[18] Uchida K (2008). Overproduction of *Escherichia coli* DNA polymerase DinB (Pol IV) inhibits replication fork progression and is lethal. Mol. Microbiol..

[19] Tan KW, Pham TM, Furukohri A, Maki H, Akiyama MT (2015). Recombinase and translesion DNA polymerase decrease the speed of replication fork progression during the DNA damage response in *Escherichia coli* cells. Nucleic Acids Res..

[20] Indiani C, McInerney P, Georgescu R, Goodman MF, O’Donnell M (2005). A sliding-clamp toolbelt binds high- and low-fidelity DNA polymerases simultaneously. Mol. Cell.

[21] Furukohri A, Goodman MF, Maki H (2008). A dynamic polymerase exchange with *Escherichia coli* DNA polymerase IV replacing DNA polymerase III on the sliding clamp. J. Biol. Chem..

[22] Ikeda M (2014). DNA polymerase IV mediates efficient and quick recovery of replication forks stalled at N2-dG adducts. Nucleic Acids Res..

[23] Bunting KA, Roe SM, Pearl LH (2003). Structural basis for recruitment of translesion DNA polymerase Pol IV/DinB to the beta-clamp. EMBO J..

[24] Mo JY, Maki H, Sekiguchi M (1991). Mutational specificity of the dnaE173 mutator associated with a defect in the catalytic subunit of DNA polymerase III of *Escherichia coli*. J. Mol. Biol..

[25] Sugaya Y, Ihara K, Masuda Y, Ohtsubo E, Maki H (2002). Hyper-processive and slower DNA chain elongation catalysed by DNA polymerase III holoenzyme purified from the dnaE173 mutator mutant of *Escherichia coli*. Genes Cells.

[26] Moriya M, Grollman AP (1993). Mutations in the mutY gene of *Escherichia coli* enhance the frequency of targeted G:C–>T:a transversions induced by a single 8-oxoguanine residue in single-stranded DNA. Mol. Gen. Genet..

[27] Pandya GA, Moriya M (1996). 1,N6-ethenodeoxyadenosine, a DNA adduct highly mutagenic in mammalian cells. Biochemistry.

[28] Yuan Q, McHenry CS (2009). Strand displacement by DNA polymerase III occurs through a tau-psi-chi link to single-stranded DNA-binding protein coating the lagging strand template. J. Biol. Chem..

[29] Furukohri A, Nishikawa Y, Akiyama MT, Maki H (2012). Interaction between *Escherichia coli* DNA polymerase IV and single-stranded DNA-binding protein is required for DNA synthesis on SSB-coated DNA. Nucleic Acids Res..

[30] Gruz P (2001). Synthetic activity of Sso DNA polymerase Y1, an archaeal DinB-like DNA polymerase, is stimulated by processivity factors proliferating cell nuclear antigen and replication factor C. J. Biol. Chem..

[31] Lenne-Samuel N, Wagner J, Etienne H, Fuchs RP (2002). The processivity factor beta controls DNA polymerase IV traffic during spontaneous mutagenesis and translesion synthesis *in vivo*. EMBO Rep..

[32] Heltzel JM, Maul RW, Scouten Ponticelli SK, Sutton MD (2009). A model for DNA polymerase switching involving a single cleft and the rim of the sliding clamp. Proc. Natl. Acad. Sci. USA.

[33] Fernandez-Leiro, R., Conrad, J., Scheres, S. H. & Lamers, M. H. cryo-EM structures of the *E. coli* replicative DNA polymerase reveal its dynamic interactions with the DNA sliding clamp, exonuclease and tau. *Elife* **4**, 10.7554/eLife.11134 (2015). 10.7554/eLife.11134 PMC4703070 26499492

[34] Maki H, Mo JY, Sekiguchi M (1991). A strong mutator effect caused by an amino acid change in the alpha subunit of DNA polymerase III of *Escherichia coli*. J. Biol. Chem..

[35] Yanagihara F, Yoshida S, Sugaya Y, Maki H (2007). The dnaE173 mutator mutation confers on the alpha subunit of *Escherichia coli* DNA polymerase III a capacity for highly processive DNA synthesis and stable binding to primer/template DNA. Genes Genet. Syst..

[36] Kath JE (2014). Polymerase exchange on single DNA molecules reveals processivity clamp control of translesion synthesis. Proc. Natl. Acad. Sci. USA.

[37] Georgescu RE (2009). Mechanism of polymerase collision release from sliding clamps on the lagging strand. EMBO J..

[38] Dohrmann PR, Manhart CM, Downey CD, McHenry CS (2011). The rate of polymerase release upon filling the gap between Okazaki fragments is inadequate to support cycling during lagging strand synthesis. J. Mol. Biol..

[39] Li X, Marians KJ (2000). Two distinct triggers for cycling of the lagging strand polymerase at the replication fork. J. Biol. Chem..

[40] Yuan Q, McHenry CS (2014). Cycling of the *E. coli* lagging strand polymerase is triggered exclusively by the availability of a new primer at the replication fork. Nucleic Acids Res..

[41] Jergic S (2013). A direct proofreader-clamp interaction stabilizes the Pol III replicase in the polymerization mode. EMBO J..

[42] Manosas M (2012). Mechanism of strand displacement synthesis by DNA replicative polymerases. Nucleic Acids Res..

[43] Ganai RA, Zhang XP, Heyer WD, Johansson E (2016). Strand displacement synthesis by yeast DNA polymerase epsilon. Nucleic Acids Res..

[44] Lupski JR, Weinstock GM (1992). Short, interspersed repetitive DNA sequences in prokaryotic genomes. J. Bacteriol..

[45] Dutra BE, Lovett ST (2006). Cis and trans-acting effects on a mutational hotspot involving a replication template switch. J. Mol. Biol..

[46] Kuban W (2005). Mutator phenotype resulting from DNA polymerase IV overproduction in *Escherichia coli*: preferential mutagenesis on the lagging strand. J. Bacteriol..

[47] Northam MR (2014). DNA polymerases zeta and Rev1 mediate error-prone bypass of non-B DNA structures. Nucleic Acids Res..

[48] Lai PJ (2016). Long inverted repeat transiently stalls DNA replication by forming hairpin structures on both leading and lagging strands. Genes Cells.

[49] Norris D, Kolodner R (1990). Interaction of a Saccharomyces cerevisiae strand exchange stimulatory factor with DNA. Biochemistry.

