# Supplementary material for: Collision with duplex DNA renders Escherichia coli DNA polymerase III holoenzyme susceptible to DNA polymerase IV-mediated polymerase switching on the sliding clamp
Source: Sci Rep. 2017 Oct 16;7:12755. doi: 10.1038/s41598-017-13080-1 (PMC5643309; doi:10.1038/s41598-017-13080-1)
Supplement: Supplementary file 1 — Supplemental Information [file 41598_2017_13080_MOESM1_ESM.pdf]

Supplementary Information for

Collision with duplex DNA renders *Escherichia coli* DNA polymerase III holoenzyme susceptible to DNA polymerase IV-mediated polymerase switching on the sliding clamp

Thanh Thi Le[†], Asako Furukohri[†,*], Masahiro Tatsumi-Akiyama and Hisaji Maki

Division of Systems Biology, Graduate School of Biological Sciences, Nara Institute of Science and Technology, Ikoma, Nara, 630-0192, Japan

*Correspondence and requests for materials should be addressed to A.F. (email: furukori@bs.naist.jp), [†]these authors contributed equally to this work.

Experiment #1

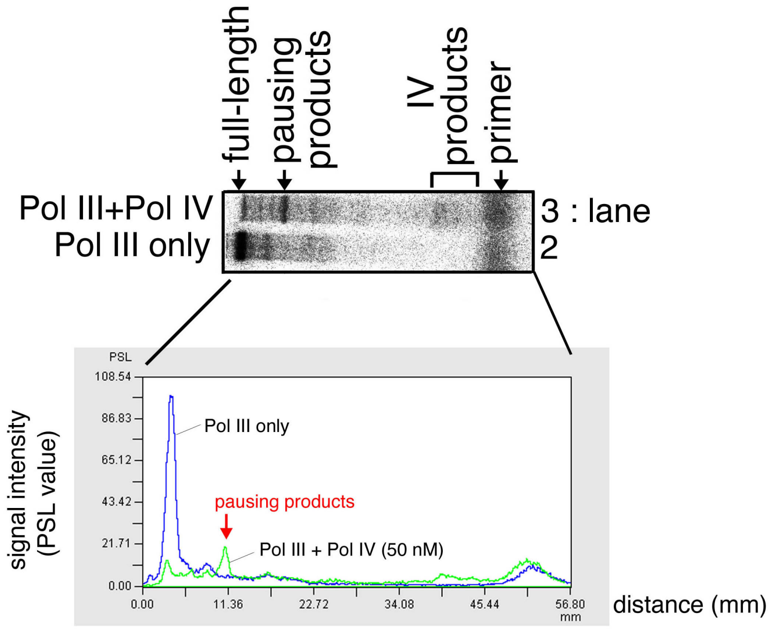

Experiment #2

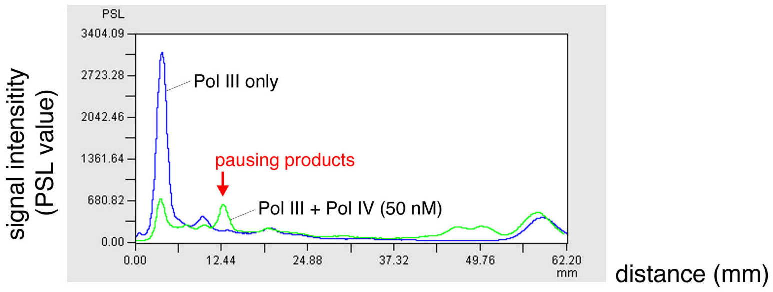

Supplemental Figure S1: The addition of Pol IV inhibits Pol III-catalyzed elongation and causes the appearance of a pausing band at around the inverted repeat.

Upper **(Experiment #1):** The effects of Pol IV on Pol III-catalyzed elongation on singly primed pMS2-aIR-23 ssDNA were quantitatively analyzed. Intensities of photostimulated lumenscence (PSL) value of radioactive signals of the part of gel image shown in Figure 1D, lanes 2 and 3 were quantified from template signals (blue line, Pol III only; green line, Pol III with wild-type Pol IV at 50 nM). The rotated corresponding image is shown in the upper panel.

Lower **(Experiment #2):** An independent experiment was carried out as in Figure 1D with wild-type Pol IV and the quantification results are similarly shown to test the reproducibility of results.

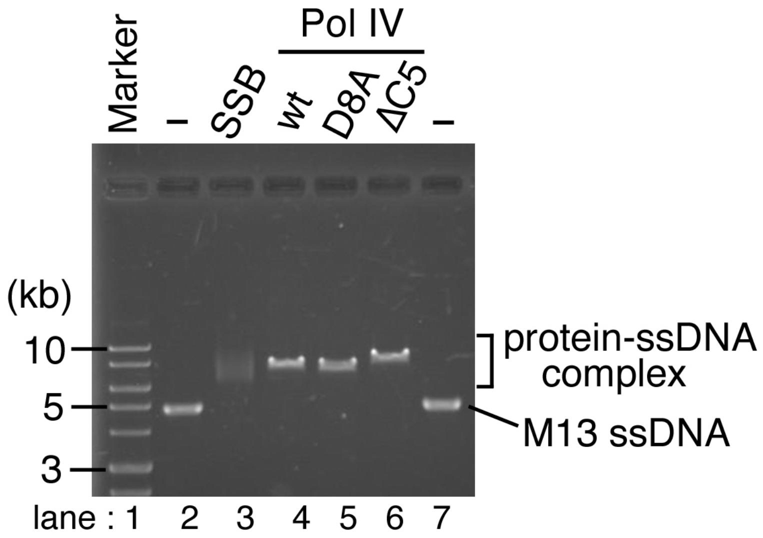

Supplemental Figure S2: Wild-type and mutant Pol IV stably bind to a circular ssDNA M13mp18.

Gel-shift analysis showing that mutant Pol IV D8A and ΔC5 bind to M13 ssDNA as well as wild-type Pol IV does. M13mp18 ssDNA was incubated with wild-type, D8A or ΔC5 mutant Pol IV for 3 min, respectively (lanes 4-6). A protein-ssDNA complex was separated on a 0.8% agarose gel electrophoresis. SSB was used as a positive control (lane 3). DNA without a protein was also shown as a control (lanes 2 and 7). See Supplemental Materials and Methods for details.

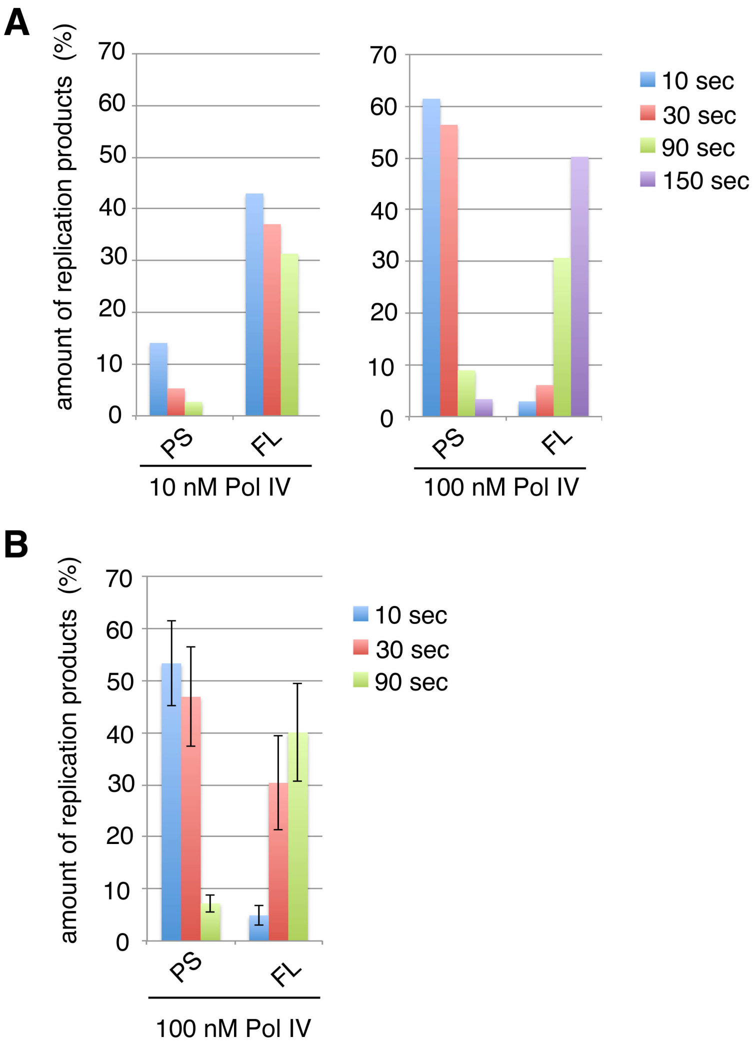

Supplemental Figure S3: Quantitative analysis of replication products by Pol III across the hairpin site in the presence of Pol IV over the time course.

A. The amount of pausing and full-length products in Figure 5B, lanes 4-6 (10 nM Pol IV) and lanes 7-10 (100 nM Pol IV) were quantified at each time point (10 – 150 seconds). The percentages of pausing products (PS) and full-length (FL) products relative to total products elongated by Pol III (>~140 nt) were calculated as in Fig 2C.

B. The time-dependent changes of pausing and full-length products were analyzed as in Figure 5B at 10, 30 and 90-second and the average percentages were calculated from two independent experiments and shown with SD.

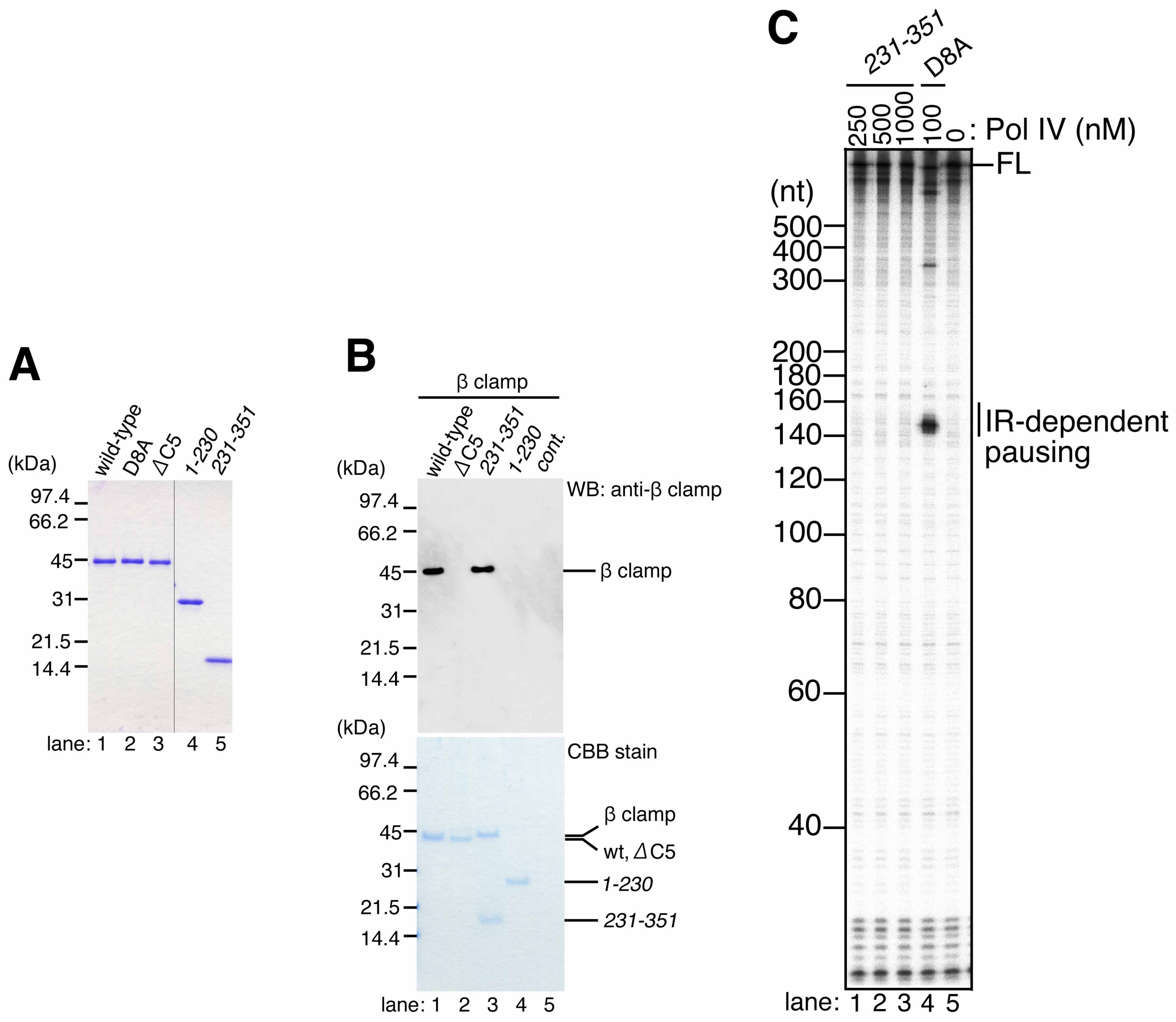

Supplemental Figure S4: The binding of Pol IV little finger domain to the β clamp is not sufficient to inhibit Pol III elongation at the inverted repeat.

A. Purified His-tagged Pol IV proteins were analyzed by the standard Laemli SDS-PAGE and Coomassie Brilliant Blue staining procedures.

B. Pol IV *231-351* containing the little finger domain (residues 231-351) binds to the β clamp as stably as wild-type Pol IV.

Pull-down assay using the β clamp and wild-type or mutant Pol IV were carried out as described under Supplemental Materials and Methods. Briefly, His-tagged Pol IV and no-tagged β clamp were incubated at 4˚C. Proteins were precipitated by Ni^{2+} magnetic beads and separated on a NuPAGE Bis-Tris 4-12% gel. The β clamp bound to Pol IV was detected by Western blotting (WB: *upper*) or Colloidal Coomassie staining (CBB:

lower). Note that wild-type Pol IV (39.5 kDa) and the β clamp (40.6 kDa) migrated at similar positions in lane 1.

C. The effect of excess amount of Pol IV *231-351* on Pol III-catalyzed elongation was tested using primed pMS2-aIR-23 as in Figure 4A.

Pol IV D8A or Pol IV *231-351* was added to the reaction mixture at a final concentration of 100 (D8A, lane 7) or 250, 500 or 1000 nM (*231-351,* lanes 1-3) together with dTTP when the Pol III-catalyzed elongation started. The replication products at 10-second incubation were analyzed as in Figure 2B. No negative effect of Pol IV *231-351* on Pol III was observed even at the highest concentration (lane 3).

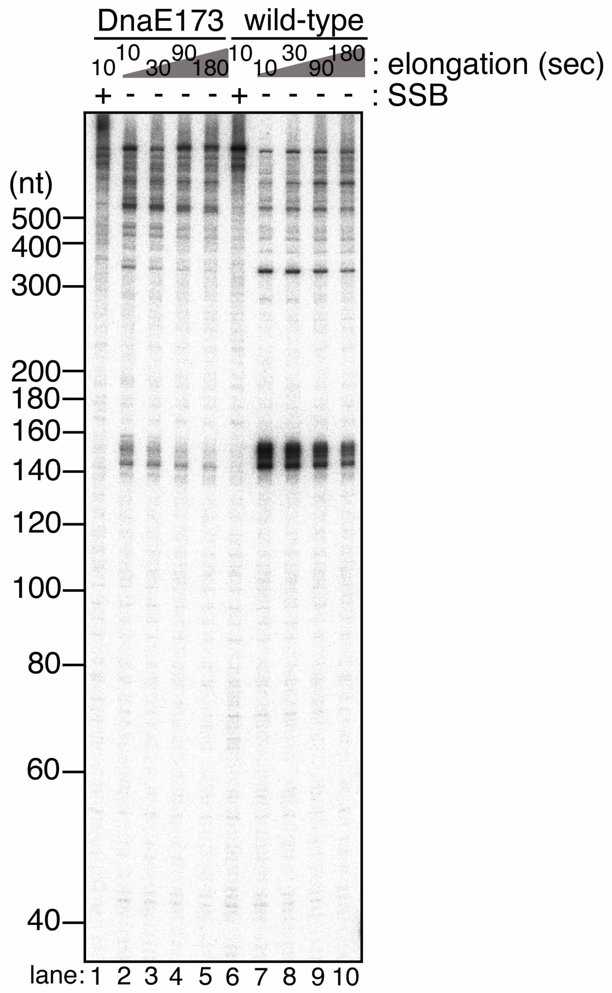

Supplemental Figure S5: Pol III$_{dnaE173}$ is more resistant to the hairpin structure formed at the inverted repeat than wild-type Pol III in the absence of Pol IV.

DNA synthesis assay in the absence of Pol IV was carried out using primed pMS2-aIR-23 as a template DNA. Replication products of wild-type or mutant Pol III in the presence or absence of SSB were analyzed at indicated time points. Pol III$_{dnaE173}$ HE overcomes the hairpin even without the aid of SSB.

Supplemental Materials and Methods

Pull-down assay

Pull-down assay was carried out with His-tagged Pol IV (wild-type, ΔC5, *1-230* and *231-351*, respectively) and the β clamp using Ni-NTA magnetic beads (QIAGEN). Pol IV (1 μg) and the β clamp (400 ng) were mixed and incubated at 4˚C for 2 hours in 25 μl of Binding Buffer (25 mM HEPES-NaOH pH 7.5, 50 mM NaCl, 20 mM Imidazole, 5% glycerol and 1 mM dithiothreitol). For the control, a buffer used in the final step of Pol IV purification was added instead of the protein (lane 5). Magnetic beads were then added to each reaction and gently mixed at 4˚C for 1 hour. The beads were corrected, washed twice with 0.5 ml Binding Buffer and bound proteins were eluted by 20 μl of Elution Buffer (Binding Buffer supplemented with 100 mM EDTA). Eluted proteins were divided into two, and each aliquot was respectively analyzed by NuPAGE 4-12% Bis-Tris gel electrophoresis (Thermo Fisher Scientific) followed by Western blotting using an antiserum against the β clamp (the generous gift from Dr T. Katayama, Kyushu University) or followed by QC Colloidal Coomassie Staining (Bio Rad).

Gel-shift assay

Pull-down assay was carried out with His-tagged Pol IV (wild-type, D8A and ΔC5) or SSB with M13mp18 circular ssDNA. Pol IV (500 ng) or SSB (300 ng) were mixed with M13mp18 (0.045 pmol) and incubated at 28˚C for 3 min in 10 μl of EDBG. The reaction mixture was mixed with 2 μl loading dye (50 mM Tris-HCl pH 7.5, 50% glycerol, 1mM EDTA, 0.1% bromophenol blue) and a protein-ssDNA complex was separated on 0.8% agarose gel in 1xTAE buffer.
